# Availability of iron ions impacts physicochemical properties and proteome of outer membrane vesicles released by *Neisseria gonorrhoeae*

**DOI:** 10.1038/s41598-023-45498-1

**Published:** 2023-10-31

**Authors:** Jagoda Płaczkiewicz, Katarzyna Gieczewska, Marcin Musiałowski, Monika Adamczyk-Popławska, Paweł Bącal, Agnieszka Kwiatek

**Affiliations:** 1 Department of Molecular Virology, Institute of Microbiology, Faculty of Biology, University of Warsaw, 02-096 Warsaw, Poland; 2 Department of Plant Anatomy and Cytology, Institute of Experimental Biology and Plant Biotechnology, Faculty of Biology, University of Warsaw, Warsaw, Poland 02-096; 3 Department of Geomicrobiology, Institute of Microbiology, Faculty of Biology, University of Warsaw, Warsaw, Poland 02-096; 4 International Centre for Translational Eye Research, Ophthalmic Biology Group, Warsaw, Poland 01-230; 5 Institute of Paleobiology, Polish Academy of Sciences, Warsaw, Poland 00-818

**Keywords:** Bacteria, Bacteriology, Pathogens

## Abstract

Outer membrane vesicles (OMVs) are bilayer structures released by bacteria for various purposes, e.g., response to environmental factors, bacterial communication, and interactions with host cells. One of the environmental variables bacteria need to react is the amount and availability of iron, a crucial element for bacteria biology. We have investigated the impact of the iron amount and availability on OMV secretion by pathogenic *Neisseria gonorrhoeae*, which, depending on the infection site, challenges different iron availability. *N. gonorrhoeae* releases OMVs in iron starvation and repletion growth environments. However, OMVs differed in physicochemical features and proteome according to iron amount and availability during the bacteria growth, as was analyzed by Liquid Chromatography-Tandem Mass Spectrometry, Infrared spectroscopy with a Fourier transform infrared spectrometer, and Atomic Force Microscopy. OMVs from iron starvation and repletion conditions had a higher variation in size, different flexibility, and different membrane protein and lipid components than OMVs isolated from control growth conditions. These OMVs also varied qualitatively and quantitatively in their total proteome composition and contained proteins unique for iron starvation and repletion conditions. Thus, the modulation of OMVs' properties seems to be a part of *N. gonorrhoeae* adaptation to surroundings and indicates a new direction of antigonococcal proceeding.

## Introduction

Outer membrane vesicles (OMVs) are small (100–300 nm), spherically bilayered structures released by Gram-negative bacteria into the extracellular milieu. OMVs comprise many cellular components, such as phospholipids, nucleic acids, cell wall components, metabolites, signaling molecules, and proteins. Owing to their cargo, OMVs participate in the biology of bacteria, including stress response, bacterial communication, and interactions with host cells, e.g., evasion of an immune response, transfer of virulence factors, and adhesion^1–4^. Several bacterial species, e.g., *Escherichia coli*, *Pseudomonas aeruginosa*, *Shigella* sp., *Salmonella* sp., *Helicobacter pylori*, *Campylobacter jejuni*, *Borrelia burgdorferi*, *Vibrio* sp., and *Neisseria meningitidis* have been reported to secrete OMVs^5–12^.

*Neisseria gonorrhoeae* (gonococcus) is an obligate human Gram-negative pathogen responsible for the second most common sexually transmitted disease: gonorrhea^13^. *N. gonorrhoeae* infects a broad array of human mucosal surfaces, most frequently related to the genitourinary tract. Untreated gonococcal infections may lead to severe complications, including pelvic inflammatory disease, ectopic pregnancy, and infertility^14,15^. Additionally, gonorrhea, with over 106 million new cases annually, is problematic due to increasing multidrug resistance, including last-line treatments like third-generation cephalosporins and azithromycin^15^. It was highlighted by the World Health Organization by listing *N. gonorrhoeae* to be a "priority pathogen".

*N. gonorrhoeae* releases OMVs, and these membrane structures contain over 200 proteins with various putative biological functions^16–18^. Already published data indicate that gonococcal OMVs influence human host cells by stimulating an immune response in non-phagocytic cells and by apoptosis induction of macrophages^17,19^. Recently, Dhital et al.^20^ demonstrated that OMVs of clinical gonococcal strains carry β-lactamase, enabling the survival of gonococci and promoting their antibiotic resistance.

Iron is a crucial element involved in bacteria's growth. However, low Fe^3+^ bioavailability (related to the low solubility of iron ions) and high reactivity of iron toward H_2_O_2_ (formation of toxic hydroxyl radicals by Fenton reaction) are harmful to live cells^21^.

The level of iron ions differs between men's and women's genitourinary tracts. To gain iron, *N. gonorrhoeae* does not encode siderophores but acquires iron from host iron-sequestering proteins: transferrin, lactoferrin, and hemoglobin. The human host responds to infection by implementing an iron starvation environment^22–24^. Therefore, *N. gonorrhoeae* has to react to stress conditions related to the availability of iron ions in colonized environments.

The response of *N. gonorrhoeae* to different availability of iron ions was yet indicated by demonstrating differential transcriptional profiles of the bacteria grown in media with different availability of iron ions. Analysis of the transcriptome of *N. gonorrhoeae* FA1090 cultured in low and high concentrations of iron ions demonstrated changes in the expression of 203 genes that encode proteins involved in various processes, including transport, metabolism, and virulence^25^*.*

We aimed to study the impact of the different availability of iron ions on the secretion and features of OMVs secreted by *N*. *gonorrhoeae* regarding that the release of OMVs is part of Gram-negative bacteria response to a changing environment. We demonstrated that *N. gonorrhoeae* modulates OMVs’ size, flexibility, protein, and lipid composition in response to iron ions availability. Such an effect may reflect the adaptation of gonococci to various environmental conditions encountered during the infection of the human host.

## Results

### *N. gonorrhoeae* MS11 releases OMVs during growth in different availability of iron ions

Previously, it was demonstrated that *N. gonorrhoeae* releases OMVs^16,17,20^; however, the iron impact on this process has yet to be evaluated. We studied the OMV secretion by *N*. *gonorrhoeae* MS11 in iron starvation and repletion conditions. To this aim, *N. gonorrhoeae* was cultivated in iron repletion conditions (GCBL medium supplemented with Fe(NO_3_)_3_) or iron starvation conditions (GCBL medium with the addition of iron ions chelator—deferoxamine). The OMVs released in the above conditions were compared to those when bacteria were cultivated in a GCBL medium (control medium, control conditions). The Methods section includes a detailed description of the media used for bacteria cultivation.

First, we measured the concentration of iron ions in culture media using Flame Atomic Absorption Spectroscopy before bacteria inoculation. Adding Fe(NO_3_)_3_ to the culture medium increased the iron ion concentration six times compared to the control medium, from 0.76 ± 0.01 to 4.67 ± 0.04 mg/ml, respectively. The addition of deferoxamine did not affect the overall Fe concentration of 0.72 ± 0.05 mg/ml, but the iron ion chelator sequesters free iron ions to form non-available for bacteria.

The iron concentration was also measured at the time of collecting the released OMVs. The iron concentrations did not vary statistically significantly between culture start and time of OMVs harvesting. At the time of OMVs collection, the iron concentration in the culture media was 0.92 ± 0.15, 0.77 ± 0.02, and 5.06 ± 0.3 mg/ml for control, iron starvation, and iron repletion conditions, respectively, (*p* > 0.05 as compared to the culture start).

**Figure 1:**
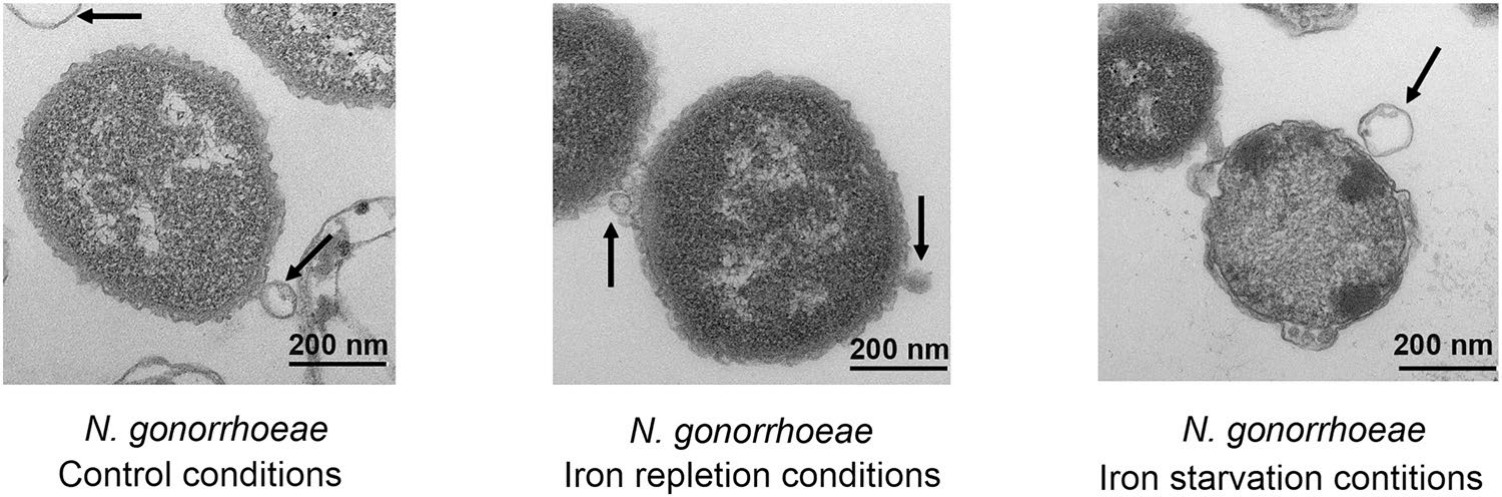
Single *N. gonorrhoeae* MS11 cells secreting OMVs, when bacteria were cultivated in a liquid media with different availability of iron ions. OMVs and bacterial cells were visualized by transmission electron microscopy (TEM). Arrows indicate budding OMVs from bacteria cells. All experiments were performed in triplicate, and representative images are shown.

As presented in Fig. 1, *N. gonorrhoeae* MS11 released OMVs while cultured in the control medium and iron repletion and starvation conditions. In all studied growth conditions, bacteria had intact cell shields, and the blebbing of the OMVs was visible.

### The iron availability during the growth of *N. gonorrhoeae* MS11 affects the size and physicochemical proprieties of released OMVs

**Figure 2:**
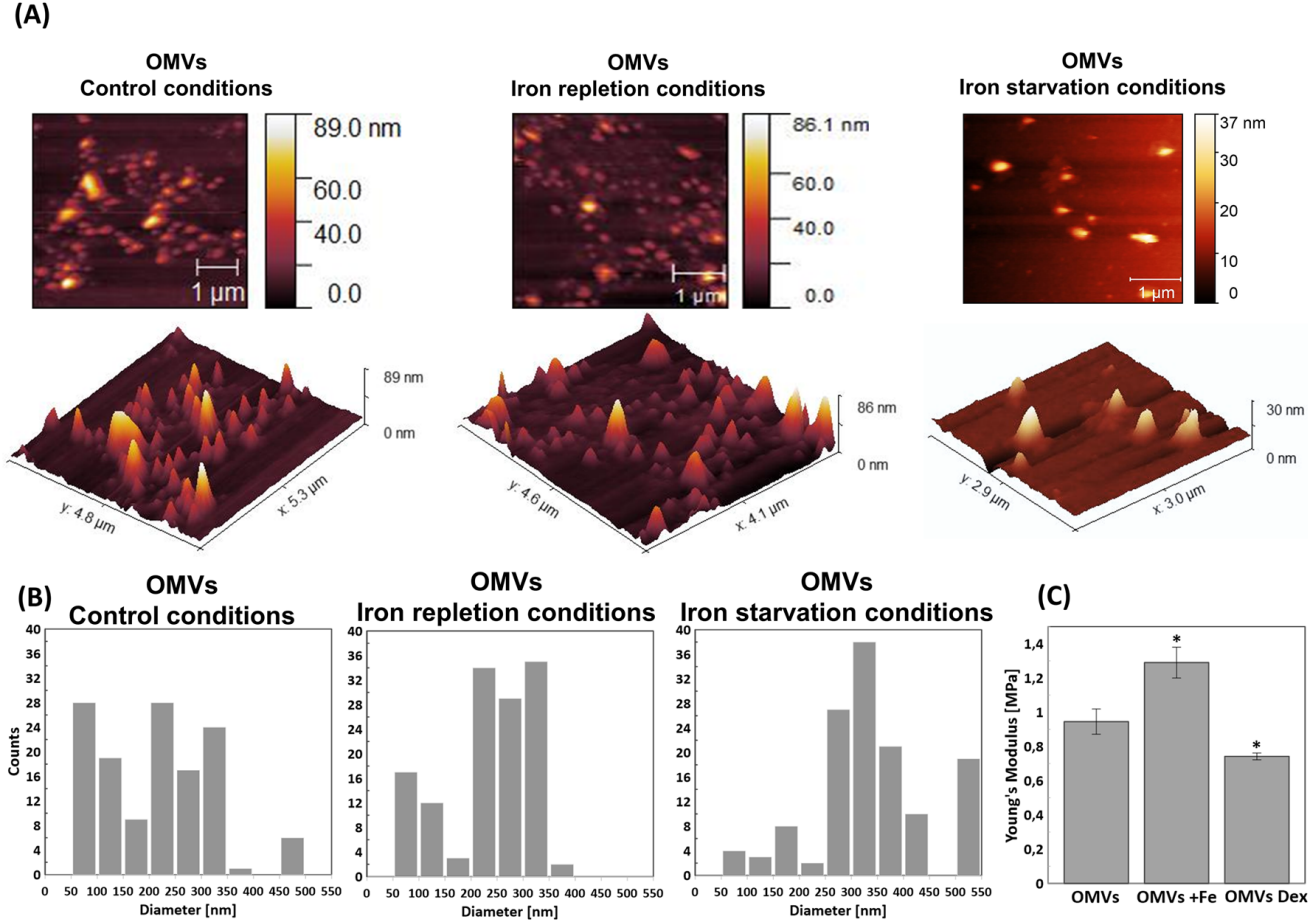
Characterization of OMVs by AFM: (**A**) Representative images of topography and 3D imaging; (**B**) histograms of OMVs diameter distributions (n > 100); (**C**) elasticity measured via Young's modulus (n > 25). Pairs of results marked with an asterisk differ significantly at *p* = 0.05 (one-way ANOVA with posthoc Tukey test). In panel (**C**), OMVs—control OMVs, Dex—OMVs released in iron starvation conditions; Dex—deferoxamine); Fe—OMVs released in iron repleted conditions; Fe—Fe(NO_3_)_3_.

TEM images of bacteria secreting OMVs suggested that the size of OMVs released in iron repletion and iron starvation conditions differs from those released in control conditions (called the control OMVs) (Fig. 1). To determine actual OMVs' sizes, secreted OMVs were separated from bacteria, purified by OptiPrep™ density gradient, and their diameters were measured by Atomic Force Microscopy (AFM). The sizes of the majority of control OMVs were in the range of 200–350 nm in diameter, but much smaller (50–100 nm) and much larger (450–500 nm) structures were also observed. The sizes of OMVs released in iron repletion conditions were less variable and were principally in the 200–350 nm diameter range. The diameters of OMVs secreted by bacteria in iron starvation conditions were 250–400 nm for most outer membrane vesicles; however, larger structures (500–550 nm in diameter) were also observed (Fig. 2A and B). Variation in the size of the secreted OMVs was also observed when the purified vesicles were visualized by TEM (Fig. S1).

Using AFM, we also measured the elastic deformation of OMVs' membrane by determination of Young's modulus. The control OMVs' elasticity expressed by Young's modulus oscillated ~ 1 MPa. We observed a significant increase in the value of Young's modulus (to 1.3 MPa) for OMVs produced in iron repletion conditions. The higher parameter indicates a stiffer OMVs’ structure, whereas the lower parameter (0.75 MPa)—as observed for OMVs produced in iron starvation conditions—the softer OMVs’ structure (Fig. 2C).

Since OMVs secreted by bacteria cultivated in iron repletion or iron starvation conditions vary in elasticity from control OMVs, we have analyzed the components of membranes of purified OMVs. For this purpose, we used Infrared spectroscopy with a Fourier transform infrared (FTIR) spectrometer. The spectrum obtained by the FTIR method is divided depending on the frequency of vibrations of the main chemical components.

**Figure 3:**
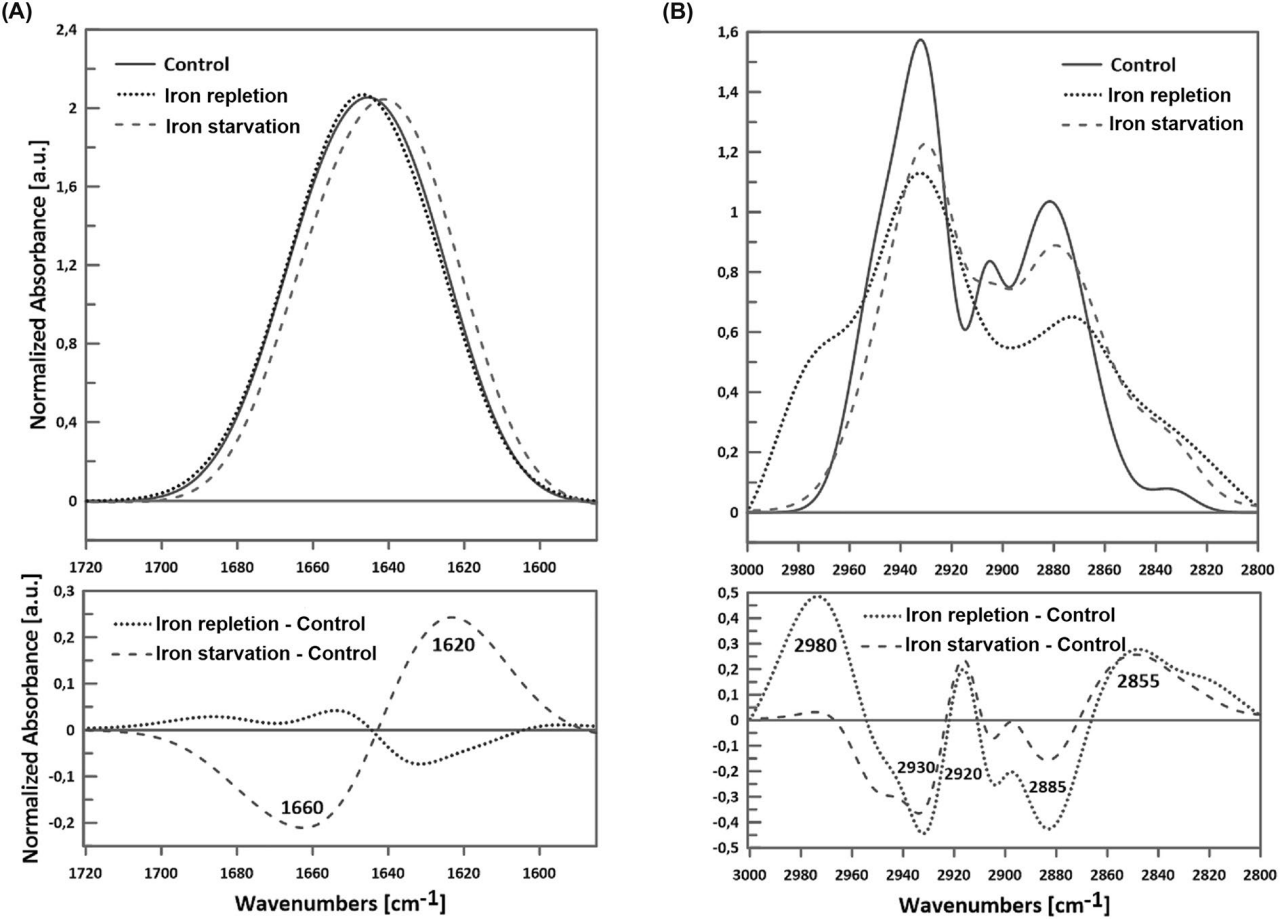
Representative spectra of OMVs' Infrared spectroscopy: (**A**) the Amide I region (upper chart) and its subtracts (lower chart); (**B**) the lipid acyl chains region (upper chart) and its subtracts (lower chart). Spectra were normalized to 100 Area Under Peak (AUP). Fe(NO_3_)_3_ and Deferoxamine stand for OMVs secreted by *N. gonorrhoeae* MS11 cultivated in iron repletion and iron starvation conditions, respectively. Control—OMVs secreted by *N. gonorrhoeae* MS11 cultivated in a control gonococcal medium.

We have analyzed the Amide I protein band 1585–1720 cm^−1^ for protein-membrane components and the C-H lipid band 2800–3000 cm^−1^ for lipid-membrane components (Fig. 3). Within the Amide I protein band, we evaluated the vibrations corresponding to the secondary structures of proteins: 1675–1695 cm^−1^ anti-parallel β-sheet aggregated structures, turns and loops 1660–1685 cm^−1^, α-helix 1648–1670 cm^−1^, β-sheet 1625–1640 cm^−1^ and aggregates 1610–1628 cm^−1^ according to rules presented in Tamm and Tatulian^26^. Analyzing lipid bands, we considered symmetrical CH_2_ groups of lipids stiffened by interaction with transmembrane helices (the so-called boundary lipids) (2825–35 cm^−1^), asymmetric and symmetrical bonds of the CH_2_ group (near 2920 and 2850 cm^−1^), symmetric vibrations of the CH_3_ group near 2870 cm^−1^ and asymmetric ones near 2960 cm^−1^ according to Derenne et al.^27^.

As is presented in Fig. 3A, the Amide I range 1585–1720 cm^−1^ differed between OMVs released by bacteria cultivated in iron starvation or iron repletion conditions compared to OMVs obtained from control growth conditions. For OMVs obtained from *N. gonorrhoeae* grown in the iron starvation conditions, we observed a significant increase in the component of approx. 1620 cm^−1^, which is responsible for β-sheets and aggregates, and a decrease in 1660 cm^−1^, a component responsible for α-helix, compared to the OMVs control. In the case of OMVs released by bacteria cultivated in iron repletion conditions, the proportion of β-sheets was lower than under the control conditions, as indicated by the decrease in the component of 1620 cm^−1^ (Fig. 3A, Table 1).

**Table 1:** Features of OMVs isolated in different availability of iron ions.

	Control conditions	Iron starvation	Iron repletion
Size (diameter)	200–350 nm, but smaller (50–100 nm) and larger (450–500 nm) structures were also observed	250–400 nm, but larger (500–550 nm) structures were also observed	Mainly 200–350 nm
Elasticity (expressed by Young's modulus)	~ 1 MPa	~ 0.75 MPa	~ 1.3 MPa
Protein-membrane components, relatively to control conditions		↑ component 1620 cm^−1^ (responsible for β-sheets and aggregates) ↓ component 1660 cm^−1^ (responsible for α-helix)	↓ component 1620 cm^−1^ (responsible for β-sheets and aggregates)
Lipid-membrane components, relatively to control conditions		↑ asymmetric CH_2_ group,	↑ proportion of CH_2_ groups and the CH_3_ group
Total number of proteins identified by LC–MS/MS	562	473	445
Number of unique proteins identified by LC–MS/MS	78	15	35

The differences between membranes of studied OMVs were also observed concerning the lipid region of the spectrum measured as the 2800–3000 cm^−1^ band (Fig. 3B). OMVs formed in the iron repletion conditions were characterized by a higher share of the components 2980, 2920, and 2855 cm^−1^ and a lower share of 2930 and 2885 cm^−1^ as compared to results observed for OMVs isolated from control growth conditions. Thus, we noticed an increase in the proportion of CH_2_ and CH_3_ groups. In turn, OMVs formed in iron starvation conditions had a more significant proportion of 2920 and 2860–65 cm^−1^ and a smaller proportion of 2940 cm^−1^ than control OMVs, indicating the increase in the asymmetric CH_2_ group in OMVs from iron starvation conditions (Table 1).

In summary, the protein and lipid membrane components, sizes, and elasticity of OMVs released by bacteria grown in iron repletion or starvation conditions vary compared to control OMVs.

### The iron availability during the growth of *N. gonorrhoeae* MS11 affects the total proteome of released OMVs

To study whether iron availability impacts total OMVs’ proteins and not only membrane-bound proteins, the proteomic analysis of purified OMVs was carried out with LC–MS/MS.

**Figure 4:**
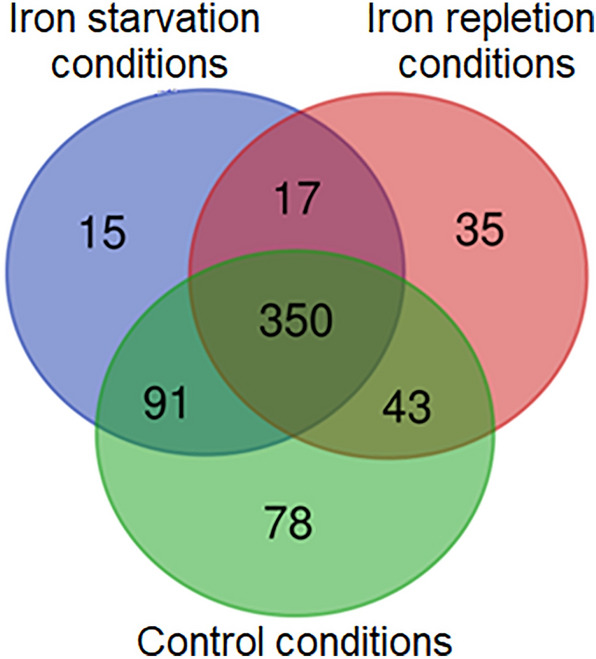
Venn diagram displaying the number of unique and common gonococcal proteins among total proteins identified in OMVs isolated from iron starvation, iron repletion, and control conditions. Numbers—the number of proteins in each group.

Four hundred forty-five proteins were identified in OMVs released by *N. gonorrhoeae* in iron repletion conditions and 473 in iron starvation conditions, compared to 562 proteins detected in the control OMVs (Table S2). Among total OMVs’ proteins, we have found 15, 35, and 78 proteins unique for OMVs from iron starvation, iron repletion, and control conditions, respectively (Fig. 4, Tables 1, 2). Three hundred fifty proteins were common for OMVs from all studied conditions. The remaining proteins belong to two kinds of studied milieu concerning iron availability (Fig. 4, Table S1).

**Table 2:** Proteins unique for OMVs from iron starvation and iron repletion conditions identified among the total proteins of the OMVs.

Protein name	Accession number	COG category
**Proteins unique for OMVs from iron starvation conditions**		
50S ribosomal protein L15	Q5F5U6	J: Translation
50S ribosomal protein L28	Q5F682	J: Translation
Hypothetical protein	Q5F555	J: Translation
Hypothetical protein	Q5F5V9	M: Cell wall/membrane/envelop biogenesis
Putative DNA ligase	Q5F595	M: Cell wall/membrane/envelop biogenesis
4-hydroxy-tetrahydrodipicolinate reductase	Q5F5Y7	E: Amino Acid metabolism and transport
Carbamoylphosphate synthase small subunit	Q5FAG9	F: Nucleotide metabolism and transport
Putative khg/kdpg 4-hydroxy-2-oxoglutarate aldolase	Q5F8Q4	G: Carbohydrate metabolism and transport
Putative 3-demethylubiquinone-9 3-methyltransferase	Q5F562	H: Coenzyme metabolism
Acyl carrier protein	Q5F604	I: Lipid metabolism
MarR-family transcriptional regulator	Q5FAG4	K: Transcription
Putative DNA polymerase I	Q5F533	L: Replication and repair
Bacterioferritin	Q5F8H9	P: Inorganic ion transport and metabolism
Hypothetical protein	Q5F713	S: Function unknown
Putative GTP-binding protein	Q5F8H0	T: Signal transduction
**Proteins unique for OMVs from iron repletion conditions**		
Putative electron transfer flavoprotein alpha-subunit	Q5F5I8	C: Energy production and conversion
Putative oxidoreductase, NAD(P)H-flavin	Q5F9K5	C: Energy production and conversion
Putative D-lactate dehydrogenase	Q5F898	C: Energy production and conversion
ATP synthase epsilon chain	Q5F4Y9	C: Energy production and conversion
ATP synthase subunit delta	Q5F4Z3	C: Energy production and conversion
NADH-quinone oxidoreductase subunit D	Q5F618	C: Energy production and conversion
Putative ribonuclease	Q5F8H2	J: Translation
50S ribosomal protein L22	Q5F5T2	J: Translation
Methionyl-tRNA formyltransferase	Q5F5P7	J: Translation
Cysteine-tRNA ligase	Q5F5D6	J: Translation
30S ribosomal protein S6	Q5F925	J: Translation
Peptide chain release factor 2	Q5F5H5	J: Translation
Integration host factor beta subunit	Q5F906	K: Transcription
Putative RpiR-family transcriptional regulator	Q5F8P9	K: Transcription
Transcription termination factor rho	Q5FA35	K: Transcription
Hypothetical protein	Q5F6N0	K: Transcription
3-isopropylmalate dehydratase large subunit	Q5F8T1	H: Coenzyme metabolism
Putative oxygen-independent coproporphyrinogen III oxidase	Q5F6H8	H: Coenzyme metabolism
Putative diaminohydroxyphosphoribosylaminopyrimidine deaminase	Q5FAD7	H: Coenzyme metabolism
Putative nicotinate-nucleotide pyrophosphorylase	Q5F6I9	H: Coenzyme metabolism
4-hydroxy-3-methylbut-2-enyl diphosphate reductase	Q5FAF2	M: Cell wall/membrane/envelop biogenesis
Elongation factor 4	Q5F9P9	M: Cell wall/membrane/envelop biogenesis
UDP-N-acetylglucosamine 1-carboxyvinyltransferase	Q5F5K6	M: Cell wall/membrane/envelop biogenesis
ATP phosphoribosyltransferase regulatory subunit	Q5F9J6	E: Amino Acid metabolism and transport
Agmatinase	Q5F6R3	E : Amino Acid metabolism and transport
Glycerol-3-phosphate dehydrogenase [NAD(P)+]	Q5F5A8	I: Lipid metabolism
Phosphate acyltransferase	Q5F4X3	I: Lipid metabolism
Hypothetical protein	Q5F507	S: Function unknown
Hypothetical protein	Q5F7D9	S: Function unknown
Putative phosphoribosylamine-glycine ligase	Q5F5I6	F: Nucleotide metabolism and transport
Hypothetical protein	Q5F4Y2	G: Carbohydrate metabolism and transport
Putative regulator of pilE expression	Q5F510	O: Post-translational modification, protein turnover, chaperone functions
Hypothetical protein	Q5F9M2	P: Inorganic ion transport and metabolism
Hypothetical protein	Q5F785	T: Signal transduction
Protein translocase subunit SecA	Q5F807	U: Intracellular trafficking and secretion

**Figure 5:**
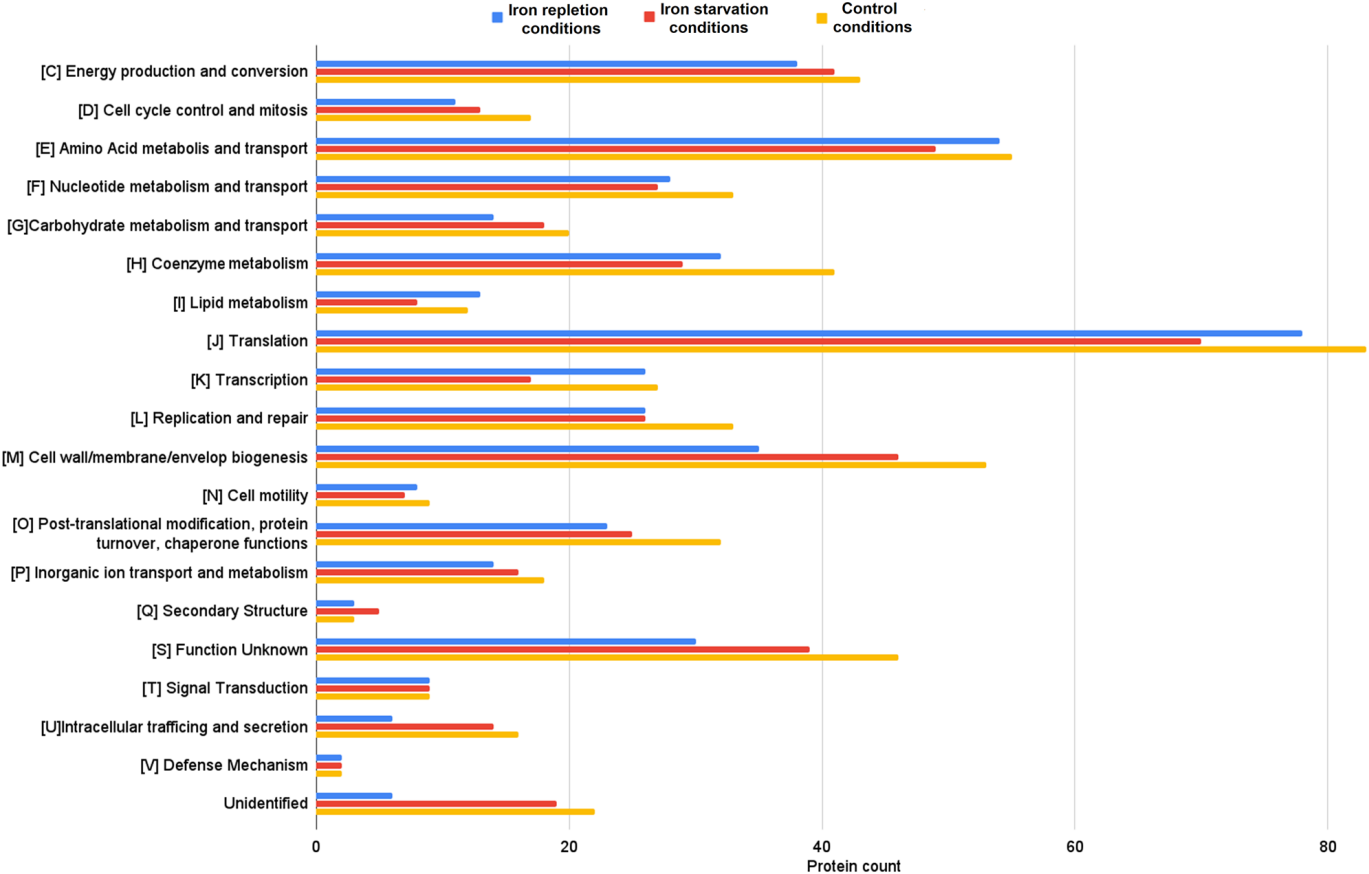
Classification of proteins identified in OMVs secreted by *N. gonorrhoeae* cultivated in media with different availabilities of iron ions according to a cluster of orthologous groups (COG). Red bars—proteins identified in OMVs secreted by gonococcus cultivated in iron starvation conditions. Blue bars—proteins identified in OMVs secreted by gonococcus cultivated in iron repletion conditions; Orange bars—proteins identified in OMVs secreted by gonococcus cultivated in a control medium. Letters represent COGs categories; numbers – represent the number of proteins in each category.

All identified OMVs’ proteins (from OMVs secreted in iron starvation, iron repletion, and control conditions) have been classified into functional categories of the cluster of orthologous groups (COG). These molecules were included in one of 20 COG categories among 26 COG categories defined in the NCBI database (Figs. 5, S2). The most represented COGs were J (Translation), E (Amino acid metabolism and transport), and M (Cell wall/membrane/envelope biogenesis) (Fig. 5). In turn, proteins unique for iron repletion or starvation conditions have been classified only in 14 or 12 COG categories, respectively (Table 2). Concerning iron repletion conditions, the most numerous COGs were C (Energy production and conversion) (6 unique proteins), J (Translation) (6 unique proteins), H (Coenzyme metabolism) (4 unique proteins), and K (Transcription) (4 unique proteins). The most numerous COGs with unique proteins identified in OMVs from iron repletion conditions were J (3 unique proteins) and M (2 unique proteins).

Thus, iron ions stress conditions (iron repletion or iron starvation) qualitatively and quantitatively affect the total proteome of secreted OMVs.

## Discussion

In the present work, for the first time, we have demonstrated that *N. gonorrhoeae* releases OMVs during growth in environments with different availability of iron ions. Subsequently, we have characterized gonococcal OMVs released in iron starvation and repletion conditions, including comparing those OMVs to the control OMVs.

For other bacteria, the OMV secretion in environments with different iron availability was demonstrated mainly for iron starvation conditions. For example, *Aeromonas salmonicida*, *Haemophilus influenzae*, *Vibrio cholerae*, *Helicobacter pylori*, *Escherichia coli,* and *Mycobacterium tuberculosis* produce OMVs in iron starvation conditions^7,28–31^. Data on OMVs released by bacteria in iron repletion conditions are minimal.

We demonstrated that different availability of iron ions in the environment leads to changes in the physicochemical features of OMVs. OMVs obtained from bacteria grown in iron starvation conditions are softer and more elastic (as shown by Young's modulus), and their membranes contain more aggregate structures and β-sheets (as indicated by FTIR). The detection of β-sheets' secondary structures may result from the presence of β-barrel proteins in the membrane (mostly pores and channels' proteins characteristic of bacterial and plastid membranes) or the close interaction of transmembrane protein domains^26,32^.

Simultaneously, we observed that the membrane of OMVs formed in starvation conditions had a more asymmetric CH_2_ group in lipids than those of control OMVs. It may increase OMVs’ membrane fluidity compared to this of control OMVs^33,34^ and can impact the rotation and diffusion of proteins and other bio-molecules within the membrane, influencing their functions^35,36^.

OMVs from iron repletion conditions are stiffer and more uniform in size, despite greater than in control conditions membrane fluidity caused by a higher content of unsaturated and shorter membranes' lipid chains. These changes in membrane properties of OMVs from environmental stress are in parallel with the data for membranes of foodborne bacterial pathogens, for which the composition and fluidity of cytoplasmic membranes is one type of adaptive response mechanism under hostile conditions^37^. In turn, Suwalsky et al. indicated that iron affects the structure of phospholipid multilayers changing their fluidity^38^.

OMVs from the iron starvation and iron repletion conditions also differed qualitatively and quantitatively in the total proteome compared to OMVs from control growth conditions. We demonstrated that for control OMVs, the most representative group of proteins was assigned to the Translation (J category), Amino acid metabolism and transport (E category), and Cell wall/membrane/envelope biogenesis (M category) COG categories. Zielke et al. and Deo et al. also classified the most numerous proteins into COG categories J and M^16,17^. Zielke et al.^16^ found 57 proteins common to OMVs of gonococcal strains FA1090, F62, MS11, and 1291. Fifty-two of these molecules were also found among the proteins of our control OMVs, including, e.g., the major outer membrane protein porin P.IB (acc. no. Q5F5V7), the LPS assembly protein LptD (acc. no. Q5F651), the 50S ribosomal protein L7/L12 (acc. no. Q5F5R4).

Since there has been no literature on gonococcal OMVs released under iron starvation and repletion conditions, we can only relate our data to those published, considering only control OMVs. As we present in Fig. 5, all proteins of OMVs from iron starvation and repletion environments are classified into 20 functional COG categories, similar to OMVs’ proteins from control growth conditions. It can indicate the engagement of these molecules in different cellular processes. However, proteins unique for iron starvation and repletion conditions were assigned only to the 14 and 12 COG categories, respectively (Tables 1 and 2). It might suggest targeting these OMVs to processes related to these COG categories, e.g., Translation (J COG category), Transcription (K COG category), or Energy production (C COG category). Consequently, proteins from these OMVs could participate in the regulation of adaptation and/or in the adjustment of bacteria to a stress response. For example, the gonococcal bacterioferritin, identified in OMVs from iron starvation conditions, could be engaged in iron metabolism. When present in the cytoplasm, this protein is involved in iron storage and protection against oxidative stress^39^. Another protein—the gonococcal putative oxidoreductase, NAD(P)H-flavin, from OMVs from iron starvation conditions is homologous to NAD(P)H-flavin oxidoreductase of *E. coli* engaged in iron metabolism^40^.

Our results also pointed out that some proteins unique for OMVs from iron starvation and repletion conditions can be putatively involved in the interactions of *N. gonorrhoeae* with the human host (Table 2). Glycerol-3-phosphate dehydrogenase (GAPDH) and elongation factor 4 could be such proteins. GAPDH is a glycolytic enzyme implicated in the pathogenesis of bacterial infections^41^. For *N. menigitidis,* mutation in *gapA-1* gene encoding GAPDH, results in decreased adhesion to human cells in vitro, suggesting a role in meningococcal pathogenesis^42^. Gonococcal GAPDH and elongation factor 4, present in OMVs separated from bacterial cells, similar to such molecules in other microorganisms^43–47^, could participate in the activation of host plasminogen. Human plasminogen is a zymogen of plasmin—a broad-spectrum protease that degrades extracellular matrix (ECM). Thus, *N. gonorrhoeae*, naturally producing low levels of proteases, owing GAPDH, could more effectively penetrate the ECM surrounding the host cell and consequently more efficiently adhere to and invade human cells. Our hypothesis on GAPDH is in line with Whitworth and Morgan, who suggest that GAPDH and OMVs work synergistically to stimulate bacterial pathogenesis^48^.

Thus, we cannot rule out that OMVs provide the above proteins to facilitate the fitness of bacteria to environmental changes and the *N. gonorrhoeae* interaction with the host in a changing environment concerning divergent iron availability.

In conclusion, our results indicate that response to iron availability, besides previously described global transcriptome changes, includes variations in the OMVs’ features. These adjustments reflect a high adaptation of gonococci to their disparate environmental niches and provide insight into the gonococcal multi-directional adaptation mechanism. That could point out new antigonococcal targets, and indicate constitutively present antigens for future vaccine development. In perspective, it is interesting to clarify the mechanism of the development of observed changes in OMVs’ properties.

## Methods

### Bacterial culture

*Neisseria gonorrhoeae* MS11 (ATCC: BAA-1833™) was grown at 37 °C and 5% CO_2_ on GC agar base (Difco, Detroit, MI, USA) supplemented with 1% Kellogg’s supplement (containing Supplement I and II), and 1% hemoglobin. For all experiments, gonococci exhibiting the same piliation and opaque phenotype were used, as determined by microscopy observation following the principles described by Dillard^49^. Before each experiment, an inoculum of a predominantly Pili^+^ Opa^+^ frozen stock of *N. gonorrhoeae* MS11 was spread and cultivated for 24 h on GC agar base with 1% Kellogg’s supplement (containing Supplement I and II) without hemoglobin to evaluate gonococcal colony morphologies under a stereo dissecting microscope. Next, Pili^+^ and opaque phenotype colonies were picked, streaked on GC agar base supplemented with hemoglobin and 1% Kellogg’s supplement (containing Supplement I and II), and cultivated for 24 h.

### Isolation of *N. gonorrhoeae* MS11 outer membrane vesicles

*N. gonorrhoeae* was cultured in: (i) medium for control growth conditions (GC liquid medium supplemented with 1% Kellogg’s supplement (containing Supplement I and II) and 0.01 M NaHCO_3_^49^ (GCBL medium, called control medium); (ii) medium for iron repletion conditions (GCBL medium supplemented with the additional presence of Fe(NO_3_)_3_ (final concentration: 12 mM)); (iii) medium for iron starvation conditions (GCBL medium supplemented with iron ion chelator Deferoxamine (Sigma Aldrich, Saint Louis, MO, USA) (final concentration: 100 µM)). All bacterial cultures were conducted in volume of 600 ml at 37 °C, at 220 RPM, to the late log phase (OD_600_ = 0.5). OMVs were isolated according to an optimized protocol for *N. gonorrhoeae*^50^. Obtained bacterial cultures were centrifuged (10,000 × *g*, 20 min, 4 °C), and supernatants from the same cultures were passed through 0.22 µm PES filter units (GenoPlast Biotech S.A., Rokocin, Poland) to remove remained cellular debris. Further, supernatants were subjected to ultracentrifugation (104,000 × *g*, 3 h, 4 °C) in a Beckman Type 45 Ti rotor (Beckman Coulter, Brea, CA, USA). To prevent proteolysis, pellets with crude OMVs were washed with PBS and suspended in PBS with Protease Inhibitor Cocktail (Sigma Aldrich). Three biological replicates of bacterial culture for each condition were performed for OMVs isolation.

### Purification of *N. gonorrhoeae* outer membrane vesicles

Crude OMVs were subjected to discontinuous OptiPrep™ (Stemcell Technologies, Vancouver, Canada) density gradient [45, 40, 35, 30, 25 & 20% (w/v)], with 1.7 ml of OMVs in 55% (w/v) density gradient medium suspension loaded at the bottom of Open-Top Thinwall Ultra-Clear, 25 × 89 mm tube, and 5.7 ml of decreasing percentage of OptiPrep™ from bottom to the top of a tube. Prepared samples were ultracentrifuged at 170,000 *g*, for 16 h, at 4 °C, using Beckman SW 32 Ti Swinging-Bucket Rotor, and 14 fractions were collected from the top, 2.5 ml each. The purity of obtained OMVs in different fractions was analyzed using Transmission electron microscopy (TEM). The concentration of obtained purified OMVs was measured based on protein content, on OMVs previously lysed with 0.2% SDS, using Bio-Rad Protein Assay (Bio-Rad, Hercules, CA, USA), and lipid content using lipophilic FM 4–64™ dye (Thermo Fisher Scientific, Waltham, MA, USA). Protein concentration was measured relative to Bovine Serum Albumin (BSA) standard protein, from 3 biological and 2 technical replicates.

### Flame atomic absorption spectroscopy (FAAS)

For quantification of iron ion concentration in a control medium and in iron repletion and starvation conditions, flame atomic absorption spectroscopy analysis was performed in the Apparatus Laboratory Instrumental Environmental Analyzes at the Faculty of Biology, University of Warsaw according to Styczyński et al.^51^. Iron ion concentration was measured using SOLAAR M Series (Thermo Fisher Scientific) and a gas mixture of air and acetylene. The calibration curve range was 0–10 mg L^−1^ and the lower limit of quantification was 0.01 mg L^−1^. For background correction, a deuterium lamp was used. The mineralization was carried out in the ETHOS Plus microwave mineralizer by Milestone (Milestone, Sorisole, Italy). The sample was dissolved in 9 ml of 69% nitric acid with the addition of 1 ml of H_2_O_2_ at 180 °C for 20 min. The concentration of the iron in samples was calculated relative to the calibration curve, from 3 biological and 2 technical replicates to obtain proper repeatability. Differences were estimated using one-way ANOVA followed by Bonferroni/Tukey posttests with a *p* value threshold set as 0.05.

### Transmission electron microscopy (TEM)

To visualize *N. gonorrhoeae* secreting OMVs, bacterial cells were fixed in 2.5% glutaraldehyde in 0.1 M cacodylate buffer pH = 7.2 overnight at room temperature. Samples were washed in cacodylate buffer three times and stained with 1% osmium tetroxide in ddH_2_O overnight at room temperature. Samples were washed in ddH_2_O and dehydrated through a graded series of ethanol (30, 50, 70, 80, 96%, absolute ethanol, and acetone). Samples were embedded in resin (SERVA) and polymerized for 24 h at 60 °C in an incubator (Agar Scientific, England). Next, 70 nm sections were cut with a diamond knife on an RMC MTXL ultramicrotome (RMC Boeckeler Instruments, USA). The sections on copper grids were contrasted with UranyLess (Micro to Nano, Netherlands) and lead citrate after 2 min^52^.

For visualization of purified OMVs, a total of 10 μL samples of OMVs solutions were absorbed onto carbon-coated 400 copper mesh grids (Microscopy Sciences, CF-400-Cu EMS, USA) for 1 min, then the excess of the solution was blotted away, and specimens were washed 3 times in droplet of distilled water, stained with 1% uranyl acetate for 1 min and air-dried.

Samples (both bacterial cells and purified OMVs) from 3 biological replicates were visualized in a LIBRA 120 transmission electron microscope (Carl Zeiss, Germany), at 120 keV. Photographs of *N. gonorrhoeae* producing OMVs and of OMVs after density gradient purification were made with a Slow-Scan CCD camera (ProScan, Germany), using the EsiVision Pro 3.2 software. Image processing was performed using the analySIS^®^ 3.0 image-analytical software (Soft Imaging Systems GmbH).

### Atomic force microscopy (AFM) measurements

AFM measurements were performed with an Agilent 5500 microscope (Agilent, Santa Clara, CA, USA), working in contact and tapping mode, under the control of PicoView software as previously described^53^. OMVs were immobilized on freshly cleaved mica, with poly-L-lysine as a stabilizing matrix. We used SNL probes (Bruker Corp., Billerica, MA, USA) with nominal k = 0.35 N/m. For elasticity determination, the k constant of each probe was measured with a built-in Agilent Thermal-K setup.

Surface probing for elasticity determination was done with a built-in plugin for PicoView software, recording and analyzing force-distance curves. Usually, a resolution of 32 × 32 was used for probing over a chosen region of about 1.5 μm × 1.5 μm. The average elasticity of pixels correlating to grains was measured in the overall image, and then the values from several images for each sample were averaged (n > 25). For AFM analysis the same amount of pure OMVs was used.

Images were analyzed with Gwydion 2.61 software^54,55^. The size of OMVs was measured by manual selection using the “line” Gwydion tool, which measures the distance between two points—at least one hundred counts were measured for each type of OMVs.

### Fourier-transform infrared (FTIR) measurements

For FTIR measurement the same amount of pure OMVs was used. Fourier-transform infrared spectra of isolated OMVs were recorded with a Nicolet iS50 FTIR spectrometer (Thermo Fisher Scientific) equipped with a single reflection diamond ATR cell with a spectral resolution of 0.6 cm^−1^. OMVs isolate was placed in the amount of 5 µl directly on the ATR crystal, dried with argon, and measured after drying. We performed two technical repetitions for samples for 3 experimental replicates. For each sample spectrum, 25 interferograms were averaged and Fourier-transformed. The spectra were normalized by subtracting the background spectrum, cutting the appropriate band, and normalizing it to 100 below the peak area. Such spectra were also used for subtraction. Data analysis was carried out with Grams/AI 8.0 Spectroscopy Software (Thermo Electron Corp., Waltham, MA, USA).

### Proteomic characterization of *N. gonorrhoeae* outer membrane vesicles by LC–MS/MS

For proteomic analysis the same amount of pure OMVs, as determined by total protein concentration, was used for analysis.

Before LC–MS/MS analysis, proteins from purified OMVs samples from 3 biological replicates for each condition were extracted with SDS (final concentration 0.2%), precipitated with methanol and chloroform, and then obtained pellets were sonicated until completely broked. Further, pellets were resuspended in HEPES 50 mM pH 8.0, sonicated, and Tris(2-carboxyethyl)phosphine (TCEP) to 5 mM and chloroacetaldehyde (CAA) to 10 mM was added. Then, 1 µg of trypsin in HEPES 50 mM pH 8.0 per 100 µg of protein was added, and samples were incubated overnight at 37 °C in a shaker (800 RPM). The reaction was quenched by the addition of formic acid (at a final concentration of 1%) and samples were kept at − 80 °C until further analysis. The samples were thawed and centrifuged at 12,000 × *g* for 3 min at room temperature. The peptides in the supernatant were desalted with the use of AttractSPE™ Disks Bio—C18 (Affinisep, cat. no. SPE-Disks-Bio-C18-100.T1.47.20) using a stage-tip protocol and dried using a Savant SpeedVac concentrator^56^. Before LC–MS measurement, the samples were resuspended in 0.1% TFA, and 2% acetonitrile in water. Chromatographic separation was performed in duplicate, for each biological replication, on an Easy-Spray Acclaim PepMap column 50 cm long × 75 µm inner diameter (Thermo Fisher Scientific) at 45 °C by applying 100 min acetonitrile gradients in 0.1% aqueous formic acid at a flow rate of 300 μl/min. An UltiMate 3000 nano-LC system was coupled to a Q Exactive HF-X mass spectrometer via an easy-spray source (all Thermo Fisher Scientific). The Q Exactive HF-X was operated in data-dependent mode with survey scans acquired at a resolution of 120,000 at m/z 200. Up to 15 of the most abundant isotope patterns with charges 2–5 from the survey scan were selected with an isolation window of 1.3 m/z and fragmented by higher-energy collision dissociation (HCD) with normalized collision energies of 27, while the dynamic exclusion was set to 30 s. The maximum ion injection times for the survey scan and the MS/MS scans (acquired with a resolution of 15,000 at m/z 200) were 45 and 22 ms, respectively. The ion target value for MS was set to 3e6 and for MS/MS to 1e5, and the minimum AGC target was set to 4.4e2.

### LC–MS/MS data processing

The data were processed with MaxQuant v. 1.6.7.0^57^, and the peptides were identified from the MS/MS spectra searched against Uniprot *Neisseria gonorrhoeae* Proteome (UP000000535) using the built-in Andromeda search engine. Cysteine carbamidomethylation was set as a fixed modification and methionine oxidation, as well as glutamine/asparagine deamidation, were set as variable modifications. For in silico digests of the reference proteome, cleavages of arginine or lysine followed by any amino acid were allowed (trypsin/P), and up to two missed cleavages were allowed. The FDR was set to 0.01 for peptides, proteins, and sites. A match between runs was enabled. Other parameters were used as pre-set in the software. Unique and razor peptides were used for quantification enabling protein grouping (razor peptides are the peptides uniquely assigned to protein groups and not to individual proteins)^53–55^.

### Bioinformatic analysis

Protein sequences were compared with GenBank on the BLAST server hosted by the National Center for Biotechnology Information (www.ncbi.nlm.nih.gov/blast), KEGG (Kyoto Encyclopedia of Genes and Genomes), and UniProt databases. The proteins identified in at least two biological replicates were considered for the bioinformatic analysis.

Functional Protein annotation was performed using eggNOG-mapper 2.1.9 server hosted by EMBL (European Molecular Biology Laboratory), using the Prodigal software algorithm^58^. Clusters of Orthologous Groups (COGs) were assigned with the NCBI Conserved Domains server (http://www.ncbi.nlm.nih.gov/Structure/cdd/wrpsb.cgi), using the COG v1.0 database.

### Statistical analysis

The observed differences were estimated and verified calculated using one- or two-way ANOVA followed by Bonferroni/Tukey post-tests with a *p* value threshold set as 0.05. Data include results from at least three experiments performed in duplicate. The protein annotation statistical score was assigned using the Prodigal software algorithm^58^.

## Chemicals and kits

Chemicals were purchased from Sigma-Aldrich unless otherwise noted. All methods with described kits were performed according to the manufacturer’s recommendations.

### Supplementary Information

Supplementary Information 1.

Supplementary Information 2.

Supplementary Information 3.

Supplementary Information 3.

## Data Availability

Proteome data are deposited at Uniprot *Neisseria gonorrhoeae* Proteome (UP000000535).
