# Supplementary figures and images for: Availability of iron ions impacts physicochemical properties and proteome of outer membrane vesicles released by Neisseria gonorrhoeae
Source: Sci Rep. 2023 Oct 31;13:18733. doi: 10.1038/s41598-023-45498-1 (PMC10618220; doi:10.1038/s41598-023-45498-1)

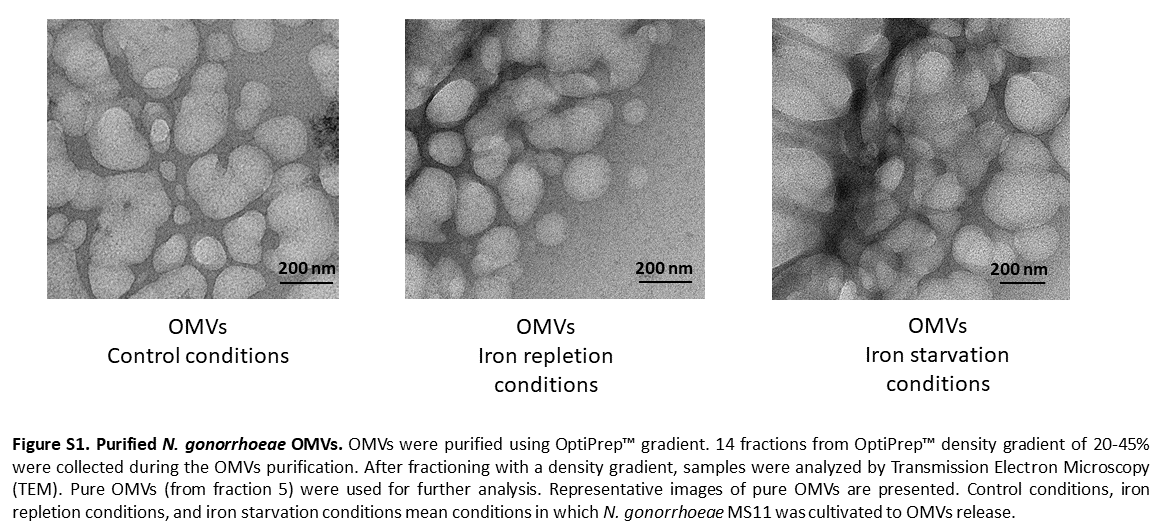

Supplement: Supplementary file 1 — Supplementary Information 1. [file 41598_2023_45498_MOESM1_ESM.tif]

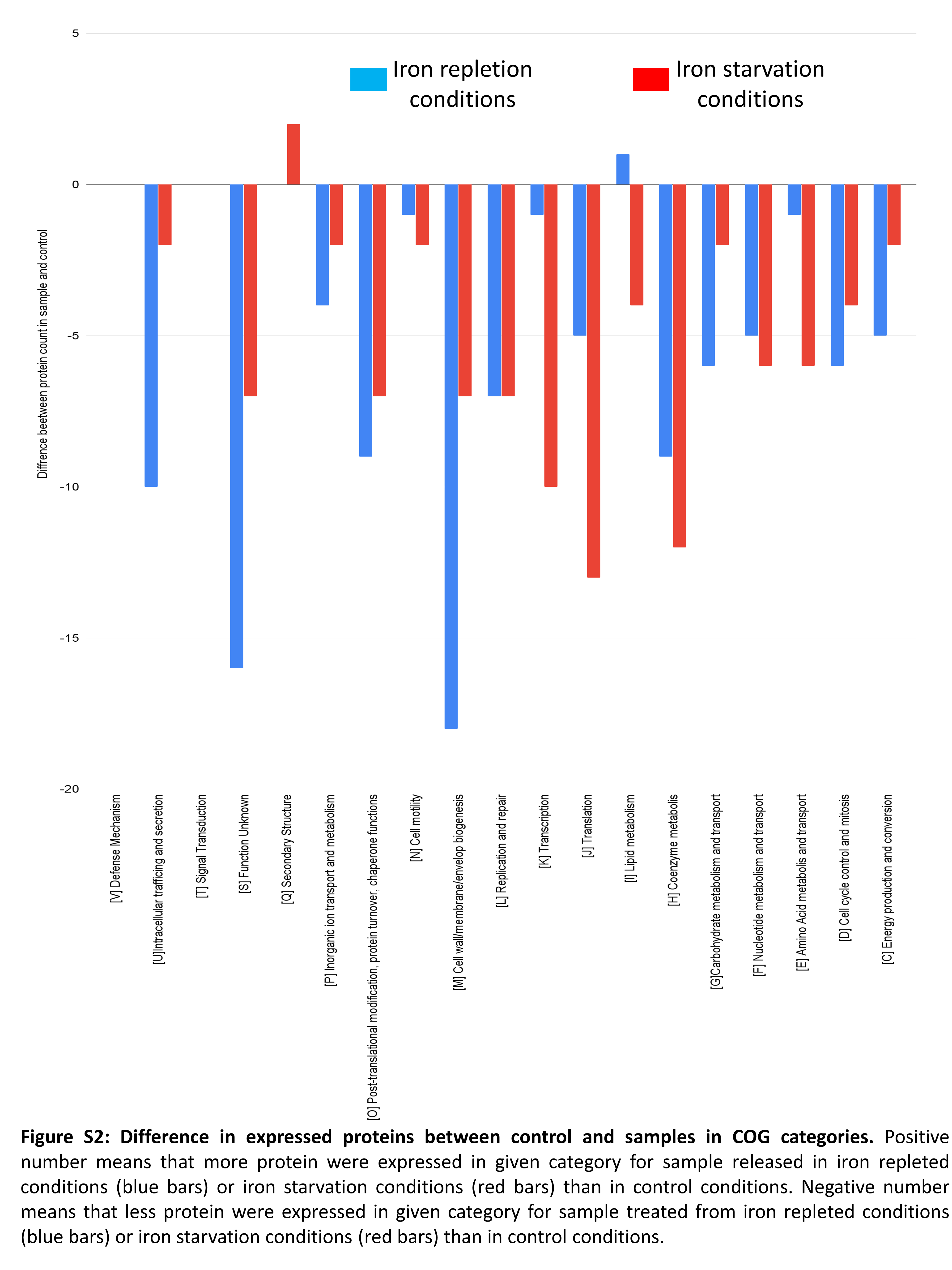

Supplement: Supplementary file 2 — Supplementary Information 2. [file 41598_2023_45498_MOESM2_ESM.tif]
