# Supplementary material for: Availability of iron ions impacts physicochemical properties and proteome of outer membrane vesicles released by Neisseria gonorrhoeae
Source: Sci Rep. 2023 Oct 31;13:18733. doi: 10.1038/s41598-023-45498-1 (PMC10618220; doi:10.1038/s41598-023-45498-1)
Supplement: Supplementary file 3 — Supplementary Information 3. [file 41598_2023_45498_MOESM3_ESM.docx]

**Table S1. Proteins of OMVs assigned to COG categories.**

| **COG category** | **Group*** | **Accession number** |
| --- | --- | --- |
| **Cell wall/membrane/envelope biogenesis (M category)** |  |  |
|  | D / F / C | Q5F7W0, Q5F9J0.1, Q5F6L6.1, Q5F569.1, Q5F501, Q5F518, Q5F5W8, Q5F651.1, Q5F6L8.1, Q5F6Q7, Q5F726, Q5F848, Q5F8G0, Q5F9V6, Q5F9W0, Q5FA01, Q5FAB9, Q5F577.1, Q5F5W3.1, Q5F5W6.1, Q5F6A4, Q5F6I1, Q5F6V5, Q5FA14, Q5FA29.1, Q5F5V7, Q5F652, Q5F7H3, Q5F932, Q5F6L9.2 |
|  | D / C | Q5F505, Q5F543, Q5F581, Q5F5H0, Q5F5W7, Q5F5Y8, Q5F6P7, Q5F6R5, Q5F7F3, Q5F8E4, Q5F8Y3, Q5F9F7, Q5F6J5, Q5F724 |
|  | F / C | Q5F845 |
|  | D | Q5F595, Q5F8L5 |
|  | F | Q5FAF2.1, Q5F5K6.1, Q5F9P9.1, Q5F584.3 |
|  | C | Q5FAC7.3, Q5F674, Q5F888, Q5F9Q9, Q5F901, Q5F584.3, Q5F531, Q5F9W2 |
| **Translation COG category (J category)** |  |  |
|  | D / F / C | Q5F685.1, Q5FA58.1, Q5F6K7.1, Q5F585.1, Q5F8K9.1, Q5F5U4.1, Q5F554.1, Q5F5Q8.1, Q5F5R2.1, Q5F5S1.1, Q5F5S3.1, Q5F5U9.1, Q5F5V0.1, Q5F6M9, Q5F8U2, Q5F8V3, Q5F902, Q5F911.1, Q5F9Q7.1, Q5FAI1.1, Q5F4Y8.1, Q5F5R4.1, Q5F5S5.1, Q5F5S8.1, Q5F5T4.1, Q5F6E8.1, Q5F6Q6.1, Q5F7C4.1, Q5F8V0.1, Q5F922.1, Q5F9R5.1, Q5FAG0, Q5F506.1, Q5F5A4.1, Q5F5F3.1, Q5F5F4.1, Q5F5R1.1, Q5F5S2.1, Q5F5T7.1, Q5F5T9.1, Q5F5V1.1, Q5F5V3.1, Q5F683.1, Q5F9G8.1, Q5F9U3.1, Q5FA41.1, Q5F4Y7.1, Q5F5S7.1, Q5F5T0.1, Q5F5W0.1, Q5F797.1, Q5FA25.1, Q5FAF5.1, Q5FAH7.1, Q5FAJ3.2, Q5F9W4.1 |
|  | D / F | Q5F5J8.1, Q5F5A5.1, Q5F5T8.1, Q5F5U2.1, Q5F7G0.2 |
|  | D / C | Q5F9T6.1, Q5F856.1, Q5F9F4.1, Q5FA57.1, Q5F5P6.1 |
|  | F / C | Q5F5U3.1, Q5F9U4.1, Q5F5Y3, Q5F835.1, Q5F905, Q5F5R3.1, Q5F5X3.1, Q5F5S9.1, Q5F938.1, Q5FA60.1, Q5F752.1 |
|  | D | Q5F682.1, Q5F555, Q5F5U6.1 |
|  | F | Q5F5P7.1, Q5F5H5.1, Q5F925.1, Q5F5T2.1, Q5F5D6.1, Q5F8H2 |
|  | C | Q5F5U1.1, Q5F4W2.1, Q5FAB4, Q5FAF7.1, Q5F7X0, Q5F8U7.1, Q5F8U8.1, Q5F686.1, Q5F923.1, Q5F9U0.2, Q5F9U1.1 |
| **Amino acid metabolism and transport COG category (E category)** |  |  |
|  | D / F / C | Q5F695.1, Q5F9Y6.1, Q5F849.1, Q5F5Z8, Q5F645.1, Q5F6U3, Q5F6Y8.1, Q5F758, Q5F7E6.1, Q5F7W8, Q5F947, Q5F967, Q5FAK8, Q5F668, Q5F6X0, Q5F761.1, Q5F812.1, Q5F847, Q5F8D4.1, Q5F8S1, Q5F8T3.1, Q5FA15.1, Q5FA24.1, Q5FA47.1, Q5FA72, Q5FAD4.1, Q5F6U0, Q5F6Y6, Q5F8W4, Q5F9F2, Q5F9Q2, Q5F5E6, Q5F7D8.1, Q5F842, Q5F9W3.1, Q5F9W7, Q5FA21.1, Q5FA23.1, Q5F7D6.2, Q5F8Q3, Q5F8B4.1, Q5F7H4, Q5F9M1 |
|  | D / F | Q5F697, Q5F7E0.1, Q5F7M1 |
|  | D / C | Q5F7L8 |
|  | F / C | Q9ZHY3.2, Q5F8C0.1, Q5F7F1, Q5F538, Q5F5G5.1 |
|  | D | Q5F5Y7.1, Q5F8D7 |
|  | F | Q5F9J6.1, Q5F6R3.1 |
|  | C | Q5FAA9.1, Q5F5C1, Q5F6F9, Q5F7E3, Q5F5D2, Q5FA22.1 |
| **Function unknown COG category (S category)** |  |  |
|  | D / F / C | Q5F9I5.1, Q5F532, Q5F5E4, Q5F5L6, Q5F654, Q5F714, Q5F773, Q5F786.1, Q5F7G3, Q5F7J0.1, Q5F7Q3, Q5F7V4, Q5F810, Q5F8X7, Q5F909, Q5F960, Q5F9H4, Q5F9I8, Q5F9W8, Q5F5P4, Q5F9S4, Q5FAD5, Q5F6F4, Q5F6I5, Q5F7Y7 |
|  | D / F | Q5F5B9, Q5F8K8 |
|  | D / C | Q5F537, Q5F5H6, Q5F5P9, Q5F6F3, Q5F765, Q5F7E1, Q5F7U9, Q5F9M5, Q5F9Y4, Q5F8M3, Q5F945 |
|  | F / C | Q5F9V2 |
|  | D | Q5F713 |
|  | F | Q5F7D9, Q5F507 |
|  | C | Q5F5N1, Q5F657, Q5F670, Q5F784, Q5F884, Q5FA09, Q5F5D9.1, Q5F8B6, Q5F8J2 |
| **Energy production and conversion COG category (C category)** |  |  |
|  | D / F/ C | Q5F619, Q5F621, Q5F684, Q5F7E7, Q5F7T8, Q5F7U0, Q5F7Y2, Q5F874, Q5F876, Q5F895, Q5FA20, Q5FAB8, Q5F4Z0.1, Q5F4Z2.1, Q5F5E9.1, Q5F5I9, Q5F6N4, Q5F731, Q5F817, Q5F817, Q5F871, Q5F8T6.1, Q5F939, Q5F5J3, Q5F601, Q5F940, Q5F6V3, Q5FA46 |
|  | D / F | Q5F942, Q5FA11, Q5F751 |
|  | D / C | Q5F759, Q5F866, Q5F8S3, Q5FAC5, Q5F8Z5, Q5F926, Q5F809, Q5F571, Q5F823, Q5F5K1 |
|  | F / C | Q5F8X6 |
|  | F | Q5F618.1, Q5F9K5, Q5F4Z3.1, Q5F5I8, Q5F898, Q5F4Y9.1 |
|  | C | Q5F4Z4.1, Q5F620, Q5F5Z2, Q5F883 |
| **Coenzyme metabolism COG category (H category)** |  |  |
|  | D / F / C | Q5F5C4.1, Q5F5X2.1, Q5F5L0, Q5F6R2, Q5F7E5.1, Q5F8L7, Q5F8X4.1, Q5F9F9.1, Q5F9K3.1, Q5F9R9.1, Q5F534.1, Q5F678.1, Q5F7N6.1, Q5F8I0.1, Q5F8Q9, Q5F9F8.1, Q5FA34, Q5FA83, Q5F824, Q5F863.1, Q5F8B3, Q5F8V6, Q5F9K8, Q5F9X9.1 |
|  | D / C | Q5F7E2, Q5F6P1.1, Q5F6E9.1 |
|  | F / C | Q5F8B4.1, Q5F7H4, Q5F5A9.2, Q5F8G6.1 |
|  | D | Q5F7D6.2, Q5F562 |
|  | F | Q5F6I9, Q5F6H8, Q5F8T1.1, Q5FAD7 |
|  | C | Q5F836.2, Q5F526, Q5F7G1, Q5F879, Q5F8V2.1, Q5FAG1.1, Q5FAH9.1, Q5F588.1, Q5F9S6.1, Q5FAC0.2 |
| **Post-translational modification, protein turnover, chaperone functions COG category (O category)** |  |  |
|  | D / F /C | Q5F541.1, Q5F6V7, Q5F771, Q5F7C9, Q5F7W9, Q5F8K6, Q5F8W5.1, Q5F9L1, Q5F7I8.1, Q5F542.1, Q5F6W5.1, Q5F6X1.1, Q5F9L7, Q5F865, Q5F8J8, Q5F9J4.1, Q5FAB3, Q5FAI6 |
|  | D / C | Q5F816.2, Q5F649, Q5F6A5, Q5F6K3, Q5F948, Q5FA91 |
|  | F / C | Q5F8E8.1, Q5F9I6, Q5F9R3, Q5F5G3 |
|  | D | Q5FA46 |
|  | F | Q5F510 |
|  | C | Q5F571, Q5F823, Q5F5K1, Q5F8V8 |
| **Cell cycle control and mitosis COG category (D category)** |  |  |
|  | D / F / C | Q5F5V4.1, Q5F915.1, Q5F6M2, Q5F6V2, Q5F8D2, Q5F933, Q5F9Z8, Q5F5X8, Q5F5K2, Q5F7W0 |
|  | D / C | Q5F5V5.1, Q5F9X1, Q5F5Y0.1 |
|  | F / C | Q5F6M3 |
|  | C | Q5F5A6, Q5F6K2, Q5FAG6 |
| **Nucleotide metabolism and transport COG category (F category)** |  |  |
|  | D / F / C | Q5F605.1, Q5F9Z5.1, Q5F5F5.1, Q5F655, Q5F7S8, Q5F878.1, Q5F8B1, Q5F8H4, Q5F8Q0.1, Q5F9X3, Q5F5P1.1, Q5F973.1, Q5F9E0.1, Q5F9P8, Q5F732.1, Q5F8Z6, Q5FAK5.1, Q5F9P0.1, Q5FAH2, Q5F6H5.2, Q5F7J4.2 |
|  | D / F | Q5F9J5.1 |
|  | D / C | Q5F5P0.1, Q5F7G6.1, Q5F5K5.1, Q5F9J3.1 |
|  | F / C | Q5F4X9.1, Q5F6T1.1, Q5F9J2.1, Q5F9Q8, Q5F7X4 |
|  | D | Q5FAG9 |
|  | F | Q5F5I6 |
|  | C | Q5F6M0.1, Q5F9Y1.1, Q5F9F5 |
| **Carbohydrate metabolism and transport COG category (G category)** |  |  |
|  | D / F / C | Q5F8Z2.1, Q5F5N5, Q5F746.1, Q5F7Y3, Q5F591, Q5F592, Q5F8T9, Q5F8L4, Q5FAI4, Q5FAJ7, Q5F8C3.1, Q5F9J0.1 |
|  | D / C | Q5F8Q1, Q5F7C0.1, Q5F8P8.1, Q5F8Q2 |
|  | F / C | Q5F8Q3 |
|  | D | Q5F7D6.2, Q5F8Q4 |
|  | F | Q5F4Y2 |
|  | C | Q5F7T9, Q5F5E3.1, Q5F9L8 |
| **Lipid metabolism COG category (I category)** |  |  |
|  | D / F /C | Q5F4X7, Q5F8Y5, Q5F4X5.1, Q5F5X0.1, Q5F696, Q5F9Y5 |
|  | D / C | Q5F887 |
|  | F / C | Q5FAH6, Q5F603, Q5F7G5 |
|  | D | Q5F604.1 |
|  | F | Q5F5A8.1, Q5F4X3.1, Q5FAF2.1, Q5F4Y0 |
|  | C | Q5F8F5.1, Q5FAH5 |
| **Transcription COG category (K category)** |  |  |
|  | D / F / C | Q5F5R5.1, Q5F890, Q5F8H6, Q5F9C0, Q5F4Z9, Q5F5E7, Q5F5R6.1, Q5F653, Q5F6W7, Q5F9Y0.1, Q5F5R0, Q5F6I0, Q5F8M0, Q5F9T5.1, Q5FA55, Q5F9K0 |
|  | F / C | Q5F798, Q5F805, Q5F5V2.1, Q5F729, Q5F7F7, Q5F777 |
|  | D | Q5FAG4 |
|  | F | Q5F6N0, Q5F8P9, Q5F906, Q5FA35 |
|  | C | Q5F792.1, Q5F6K6.1, Q5F5A2, Q5F6Y5, Q5F7J5 |
| **Replication and repair COG category (L category)** |  |  |
|  | D / F /C | Q5FAJ2.1, Q5F5D5, Q5F5Q4, Q5F6J3, Q5F6P5, Q5F720, Q5F8B2, Q5F9N9, Q5FA90, Q5F8Y0, Q5FAJ1, Q5F8J6, Q5F931.1, Q5F5Q6, Q5F5Z6, Q5F8K1, Q5F8R7, Q5F9E7, Q5F9S2, Q5FA73, Q5FAH8.1, Q5F5Q2 |
|  | D / F | O87408.1 |
|  | D / C | Q5F5M0, Q5F7J7 |
|  | F / C | Q5F5D7, Q5F9Z9.1, Q5F7H1 |
|  | D | Q5F533 |
|  | C | Q5F7H0, Q5F9P4, Q5F7B9, Q5F8W0, Q5FA80, Q5F8M9.1 |
| **Cell motility COG category (N category)** |  |  |
|  | D / F / C | Q5F8L5, Q5F9E3, Q5F9P7, Q5FAC8, Q5FAG7, Q5F5L5, Q5F912 |
|  | F / C | Q5FAD1 |
|  | C | Q5F689 |
| **Inorganic ion transport and metabolism COG category (P category)** |  |  |
|  | D / F / C | Q5F8L5, Q5F536.1, Q5F5G8, Q5F768, Q5F5Y9, Q5F6Q5, Q5F930, Q5F9M4, Q5FA17, Q5FA85, Q5F6Q4 |
|  | D / C | Q5F7W6, Q5F574, Q5FA28, Q5FA63 |
|  | F / C | Q5F7X4, Q5F8H8 |
|  | D | Q5F8H9 |
|  | F | Q5F9M2 |
|  | C | Q5F7C5 |
| **Secondary Structure COG category (Q category)** |  |  |
|  | D / F / C | Q5F7G5, Q5F5J1 |
|  | D / F | Q5F4Y0 |
|  | D / C | Q5F520 |
|  | D | Q5F604.1 |
| **Signal Transduction COG category (T category)** |  |  |
|  | D / F /C | Q5F777, Q5F707, Q5F8L5, Q5F5Q1, Q5F755, Q5F839, Q5FAJ6 |
|  | F / C | Q5F9M1 |
|  | D | Q5F8H0, Q5F7D6.2 |
|  | F | Q5F785 |
|  | C | Q5F7L8 |
| **Intracellular trafficing and secretion COG category (U category)** |  |  |
|  | D / F / C | Q5F5Q2, Q5F8L5, Q5F5G3, Q5FAD2.1, Q5F4W6.1 |
|  | D / C | Q5F531, Q5F9E3, Q5F9P7, Q5FAC8, Q5FAG7, Q5F5L5, Q5F912, Q5FAB1.1 |
|  | D | Q5F9W2 |
|  | F | Q5F807.1 |
|  | C | Q5FAD1, Q5F570, Q5F689 |
| **Defense Mechanism COG category (V category)** |  |  |
|  | D / F / C | Q5F9I9, Q5F7A6 |
| **Unidentified** | D / F / C | Q5F5A7, Q5F673, Q5F7T1, Q5F8R2, Q5F9H8, Q5F9T4 |
|  | D / C | Q5F576, Q5F5C7, Q5F5E8, Q5F5F6.1, Q5F5I3, Q5F5J7, Q5F6H4.1, Q5F7C7, Q5F7X2, Q5F8J0, Q5F8S0, Q5F8T2, Q5F9I1 |
|  | C | Q5F665, Q5F7F5, Q5F9N5 |

* D – proteins of OMVs released by *N. gonorrhoeae* cultivated in the iron starvation conditions (deferoxamine was added to a control medium)

F - proteins of OMVs released by *N. gonorrhoeae* cultivated in the iron repletion conditions (additional (FeNO_3_)_3_ was added to a control medium)

C – proteins of OMVs released by *N. gonorrhoeae* cultivated in the control conditions
